# Twenty-four-hour versus clinic blood pressure levels as predictors of long-term cardiovascular and renal disease outcomes among African Americans

**DOI:** 10.1038/s41598-020-68466-5

**Published:** 2020-07-15

**Authors:** Srividya Kidambi, Tao Wang, Thomas Chelius, Irene Nunuk, Priyanka Agarwal, Purushottam Laud, David Mattson, Allen W. Cowley, Mingyu Liang, Theodore Kotchen

**Affiliations:** Medical College of Wisconsin, 8701 Watertown Plank Road, Milwaukee, WI 53226 USA

**Keywords:** Outcomes research, Risk factors

## Abstract

In Caucasian and Asian populations, evidence suggests that 24-h blood pressures (BP) are more predictive of long-term cardiovascular events than clinic BP. However, few long-term studies have evaluated the predictive value of 24-h BP phenotypes (24-h, daytime, nighttime) among African Americans (AA). The purpose of this study is to evaluate the added value of 24-h BP phenotypes compared to clinic BP in predicting the subsequent fatal and non-fatal cardiovascular/renal disease events in AA subjects. AA subjects (n = 270) were initially studied between 1994 and 2006 and standardized clinic BP measurements were obtained during screening procedures for a 3-day inpatient clinical study during which 24-h BP measurements were obtained. To assess the subsequent incidence of cardiovascular and renal disease events, follow-up information was obtained and confirmed by review of paper and electronic medical records between 2015 and 2017. During a mean follow-up of 14 ± 4 years, 50 subjects had one or more fatal or non-fatal cardiovascular/renal disease events. After adjustment for covariates, clinic systolic and diastolic BP were strongly associated with cardiovascular/renal disease events and all-cause mortality (*p* < 0.0001). Twenty-four-hour BP phenotypes conferred a small incremental advantage over clinic BP in predicting cardiovascular/renal events, which was limited to making a difference of one predicted event in 250–1,000 predictions depending on the 24-h BP phenotype. Nocturnal BP was no more predictive than the other 24-h BP phenotypes. In AA, 24-h BP monitoring provides limited added value as a predictor of cardiovascular/renal disease events. Larger studies are needed in AA to confirm these findings.

## Introduction

Since the introduction in the 1960s of non-invasive 24-h ambulatory blood pressure monitoring (ABPM), automated devices have been refined, and ABPM has proven to be a useful strategy for detecting, confirming, and monitoring hypertension (HTN)^1–7^. One of the main advantages of ABPM over the clinic blood pressure (BP) measurements is its ability to track BP changes occurring in daily life conditions and for 24 h, thus allowing assessment of overall BP variability as well as identification of its specific components, such as nocturnal HTN and altered daytime to nighttime BP profiles (i.e. morning BP rise and non-dipping pattern of BP)^8–12^. This strategy encourages greater involvement of patients in their own care and has led to the identification and clinical significance of white coat HTN and masked HTN^13–16^.

In addition to being valuable in diagnosing HTN and monitoring BPs, 24-h BP phenotypes (24-h BP, daytime BP, and nighttime BP) have been demonstrated to be more robust predictors of development of target-organ damage and long-term cardiovascular disease (CVD) events than one-time office measurements of BP^17–21^. Moreover, nighttime BP is reportedly a stronger predictor of CVD events and other target-organ damage than either office or daytime BP^22,23^. However, there is paucity of data relating 24-h BP to clinical outcomes in African Americans with majority of longitudinal ABPM studies conducted in Caucasian and Asian populations^24–26^. HTN is highly prevalent among African Americans with higher prevalence of end-organ damage at younger ages. African Americans also have an attenuated “dip” of nighttime BP^27,28^. Consequently, diurnal BP variations may be a less discriminating predictor of CVD events in African Americans than in other racial/ethnic groups.

The purpose of this study is to compare the relationships of an ‘office/clinic’ measurement of BP and 24-h BPs with the subsequent incidence of fatal and non-fatal cardiovascular (CV) and renal disease events in an African American cohort.

## Methods

African American subjects (ages 18–55 years) were recruited for genetic studies of HTN between years 1994 and 2006 in Milwaukee by referrals, advertisements, mailings to targeted zip codes, community health fairs, screenings, and word of mouth. Subjects were defined as being African American on the basis of self-identification, birth in the continental United States, both parents reported as being African American, and English as the native language Exclusion criteria included diabetes mellitus (DM), serum creatinine > 2.2 mg/dL, body mass index > 36 kg/m^2^, and pregnancy. DM was defined by a fasting blood glucose ≥ 126 mg/dL or being on treatment for DM. All volunteers were invited to a research clinic (referred to as clinic visit throughout this manuscript) for consenting, brief phenotyping, and blood draw. All participants completed a health history questionnaire. Other clinic measures included anthropomorphic measures and routine blood chemistries. Standardized measurements of BP were obtained with subjects seated quietly for at least 10 min. Two measurements were obtained in each arm by a trained and certified observer, 2 min apart, with a sphygmomanometer, and using an appropriate cuff size for arm girth. The defining BP was the average of the 4 measurements. Of the 3,943 volunteers, 55% were female and 52% were hypertensive (defined at the time as BP > 140/90 mmHg or taking antihypertensive medications).

A subset of this cohort was invited to participate in a longer in-patient study. They were admitted to an in-patient Clinical Research Center (CRC) for an extensive 2-day phenotyping protocol. Additional exclusion criteria included known cardiovascular disease including strokes and peripheral vascular disease, active malignancies, and systolic/diastolic BP > 170/110 mmHg while receiving antihypertensive therapy. Before the in-patient study, antihypertensive drugs were withdrawn for at least 1 week, and lipid lowering medications were withdrawn for 1 month. Subjects were admitted the afternoon before the Day 1 of the protocol and were placed on a weight maintaining 150 mEq sodium and 80 mEq potassium diet. On Day 1, fasting blood was drawn for routine chemistries, including measurements of lipids, glucose, and insulin. Plasma renin activity (PRA) and serum aldosterone were measured after being supine for 60 min and again after standing for 10 min. Methods of measurement of blood chemistries and hormones have been described previously^29–31^. Beginning on Day 1, over a 24-h period, BP was measured every 20 min during the daytime (5 am–11 pm) and every 45 min during the nighttime (11 pm–5 am), with an Accutracker monitor (SunTech Medical Instruments Inc, Raleigh, NC). Subjects were included when at least 75% of the readings were valid (systolic BP readings > 250 mmHg or < 70 mmHg and diastolic BP readings > 150 mmHg and < 40 mmHg, and pulse pressures < 10 mmHg were automatically discarded) and available in each time-frame.

To assess the subsequent incidence of CVD events, follow-up information was obtained by history during a single visit to the translational research unit (TRU) when subjects could be located and consented (and verified by medical records), by National Death Index (cause of death verified by medical records), or by review of electronic medical records conducted between 2015 and 2017. The composite endpoint included morbidity and/or mortality from myocardial infarction, coronary revascularization, dissecting aortic aneurysm, congestive heart failure requiring recurrent hospitalization, stroke, and stage 5 chronic kidney disease. The criteria used to designate endpoints are shown in Supplemental Table 1. All endpoints were initially verified by two board-certified physicians and further adjudicated by a board-certified cardiologist. Those subjects who did not have any of the above endpoints for at least 10 years during the follow-up period are considered to have no CV/renal outcome.

**Table 1:** Comparison of baseline characteristics of participants with and without cardiovascular/renal disease outcomes (unadjusted).

	Overall (n = 270)	Cases (n = 50)	Controls (n = 220)
Women (%)^a^	148 (55%)	19 (38%)	129 (59%)**
Hypertensive (%)^a^	149 (55%)	45 (90%)	104 (47%)****
On anti-hypertensives (%)^a^	84 (31%)	27 (54%)	57 (26%)****
Mean length of follow-up (years)	14.0 ± 4.1	14.9 ± 5.1	13.8 ± 3.8
Age (years)	43 ± 7	45.8 ± 7.3	42.8 ± 7.0**
BMI (kg/m^2^)	28.6 ± 4.9	29.3 ± 5.7	28.5 ± 4.6
Total cholesterol (mg/dL)	179 ± 38	190 ± 37	177 ± 38*
LDL-C (mg/dL)	115 ± 36	122 ± 36	113 ± 36
HDL-C (mg/dL)	47 ± 17	44 ± 13	48 ± 18
Triglycerides (mg/dL)	97 ± 63	110 ± 57	94 ± 64
Glucose (mg/dL)	90 ± 17	92 ± 15	90 ± 18
Insulin (µIU/L)	12.5 ± 6.8	12.5 ± 6.5	12.5 ± 6.8
Creatinine (mg/dL)	0.9 ± 0.2	1.0 ± 0.2	0.9 ± 0.2**
Supine PRA (ng/mL/h)	1.0 ± 1.8	0.8 ± 1.3	1.0 ± 1.9
Standing PRA (ng/mL/h)	1.4 ± 2.7	1.6 ± 3.8	1.4 ± 2.4
Supine aldosterone (ng/dL)	4.8 ± 3.6	5.1 ± 3.4	4.7 ± 3.7
Standing aldosterone (ng/dL)	7.5 ± 5.0	8.3 ± 4.6	7.2 ± 5.1

All values are expressed as mean ± SD unless otherwise specified. Conversion to SI units (multiplication factor): total cholesterol, LDL-C, and HDL-C: 0.0259 (mmol/L), HDL-C, triglycerides: 0.0113 (mmol/L), glucose: 0.05555 (mmol/L), insulin: 6.945 (pmol/L), creatinine: 88.4 (µmol/L), PRA (pg/mL): 0.0237 (pmol/L), aldosterone: 27.74 (pmol/L).

*SD* standard deviation, *BMI* body mass index, *HDL-C* high-density lipoprotein-cholesterol, *LDL-C* low-density lipoprotein cholesterol, *PRA* plasma renin activity.

*p < 0.05; **p ≤ 0.01; ***p ≤ 0.001; ****p ≤ 0.0001.

^a^Expressed as percentages.

Separate informed consent forms were signed by all subjects participating in the baseline and follow-up outpatient and inpatient research study visits. The institutional review board (IRB) waived the necessity of signed consent for review of electronic medical records for those who could not be reached in person. All protocols were approved by the Medical College of Wisconsin and Froedtert Hospital IRB.

Cardiovascular and renal-disease related outcomes was defined as a binary variable. Bivariate analyses utilized Student’s *t* test for continuous variables, Pearson’s chi-squared test for discrete variables, and Mann–Whitney’s *U* test for median follow-up time. Logistic regression models were used to test for association of various BP measurements with all-cause mortality and cardiovascular/renal outcomes. Cox proportional hazards models were also used to test for association of various blood pressure measurements with time to all-cause mortality. Each BP measurement was treated as a continuous variable and was tested separately with the adjustment for patient’s age (categorized by ‘ < 40’, ‘40–50’ and ‘ ≥ 50’), gender, body mass index (BMI) (categorized by ‘ < 25’, ‘25–30’ and ‘ > 30’) and follow-up time (categorized by quartiles). C-statistics were used to measure the goodness-of-fit of the models. Likelihood ratio statistics were also used to test for the add-on effect between a pair of BP measurements. All *p* values are 2-sided. SAS version 9.4 (SAS Institute, Cary, NC) was used for all the analyses.

## Results

Follow-up data on CV/renal disease and all-cause mortality were obtained for a total of 270 participants in whom 24-h BPs were measured. Those with at least one CV/renal disease event (morbidity and/or mortality) are referred to as cases and those without any of these events after at least a 10-year follow-up period are referred to as controls throughout this manuscript. Among the 50 cases, 72% were cardiac events (ischemic heart disease and/or congestive heart failure) and 16% were renal complications (end-stage renal disease, hemodialysis, and/or renal transplant related to HTN). The remaining events included cerebrovascular events (52%), peripheral vascular disease (10%), and aortic dissection (6%). Forty-six percent of cases had more than one event (Supplemental Table 2). A total of 26 participants died from all causes (all-cause mortality), of which 12 were from CV/renal disease hence were part of the 50 cases mentioned above as well. Median length of follow-up was 12 years for the entire cohort (mean length of follow-up: 14.0 ± 4 years).

**Table 2:** Blood pressure levels (mean ± SD) in the all-participant cohort at baseline.

	Overall (n = 270)	Cases (n = 50)	Controls (n = 220)
Clinic SBP (mm Hg)	132 ± 22	149 ± 19	128 ± 21****
Clinic DBP (mm Hg)	86 ± 15	98 ± 15	84 ± 14****
24-h SBP (mm Hg)	130 ± 19	146 ± 15	126 ± 17****
24-h DBP (mm Hg)	78 ± 12	88 ± 11	76 ± 11****
Day-time SBP (mm Hg)	130 ± 19	146 ± 16	127 ± 17****
Day-time DBP (mm Hg)	79 ± 12	87 ± 11	77 ± 11****
Night-time SBP (mm Hg)	125 ± 20	139 ± 19	121 ± 19****
Night-time DBP (mm Hg)	72 ± 13	80 ± 14	72 ± 12***
Day–night SBP difference (mm Hg)	6 ± 9	7 ± 12	6 ± 8
Day–night DBP difference (mm Hg)	5 ± 6	7 ± 9	5 ± 6
Day–night SBP difference (%)	4.7 ± 6.6	4.5 ± 8.8	4.7 ± 6.1
Day–night DBP difference (%)	6.6 ± 8.1	7.6 ± 11.1	6.4 ± 7.2
Day–night SBP ratio	0.95 ± 0.07	0.96 ± 0.09	0.95 ± 0.06
Day–night DBP ratio	0.93 ± 0.08	0.92 ± 0.11	0.94 ± 0.07

*SD* standard deviation, *SBP* systolic blood pressure, *DBP* diastolic blood pressure.

***p ≤ 0.001; ****p ≤ 0.0001.

Baseline characteristics (unadjusted) of all participants are shown in Table 1. Comparing the two groups, cases were older and had more men. Higher percentage of cases were hypertensive (90% vs. 47%) and were on anti-hypertensive medications (54% vs. 26%) compared to controls. After adjusting for age and sex, there were no differences in BMI, serum creatinine, glucose, insulin, lipids, aldosterone, and/or PRA levels.

**Figure 1:**
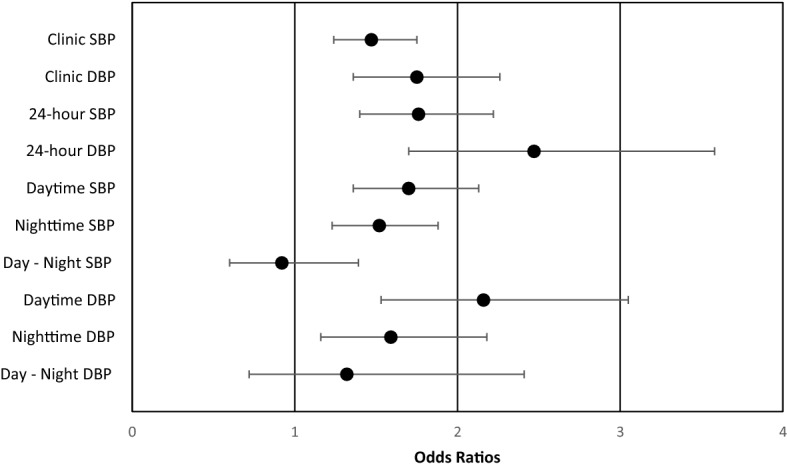
Forest plot showing odds ratio (CI) for a composite CV/renal event with different clinic and 24-h blood pressure phenotypes in the all-participant cohort. Odds ratio for compositie CV/renal events are shown for different blood pressure phenotypes are shown with overlapping CI in the all-participant cohort. *CI* confidence interval, *CV* cardiovascular, *SBP* systolic blood pressure, *DBP* diastolic blood pressure.

**Figure 2:**
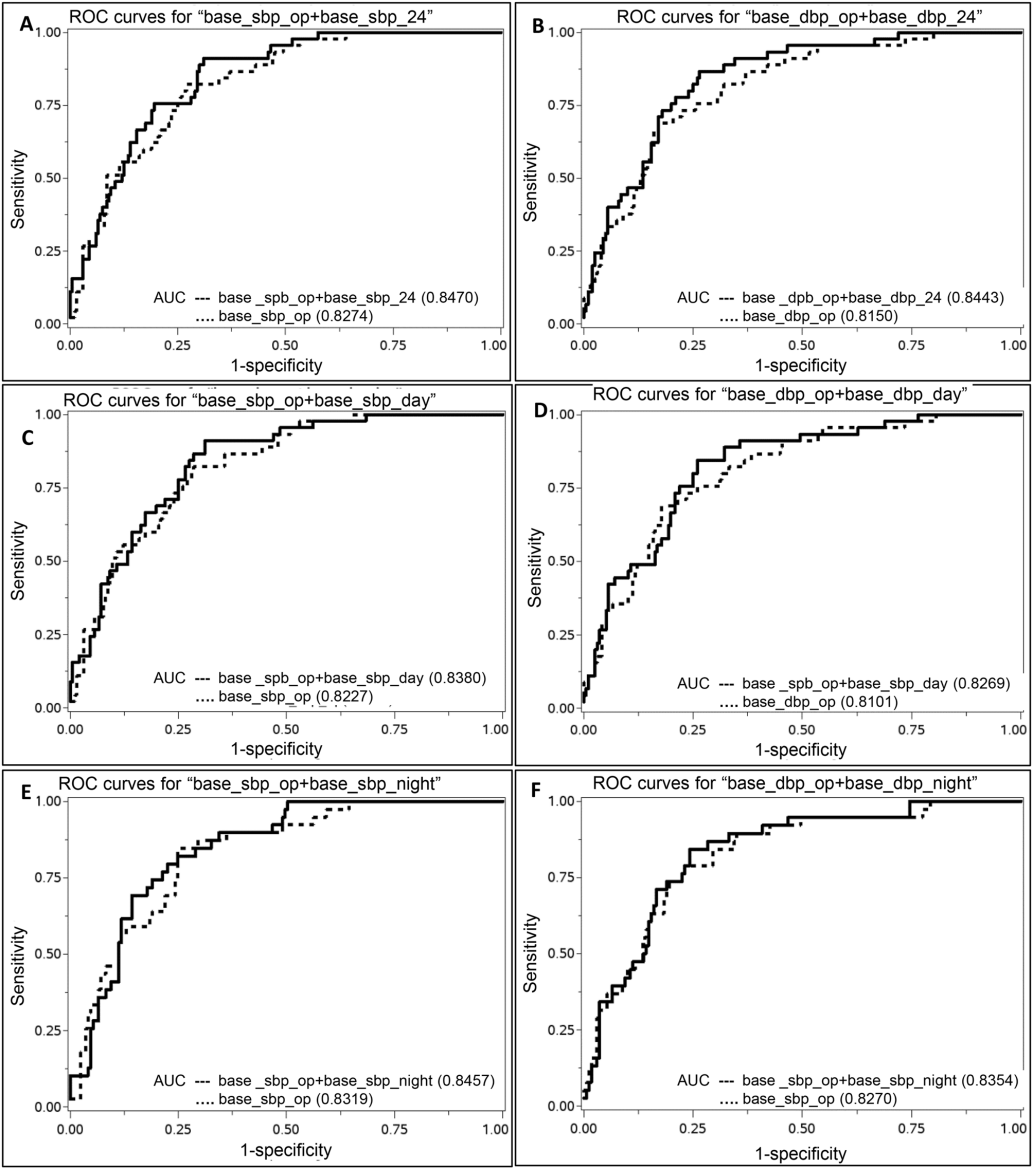
(**A**)–(**F**) Receiver-operating curves for the prediction of composite CV/renal disease events (clinic BP vs. 24-h BP phenotypes) in the all-participant cohort. (**A**) clinic SBP versus clinic SBP + 24-h SBP, (**B**) clinic DBP versus clinic DBP + 24-h DBP, (**C**) clinic SBP versus clinic SBP + daytime SBP, (**D**) clinic DBP versus clinic DBP + daytime DBP, (**E**) clinic SBP versus clinic SBP + nighttime SBP, (**F**) clinic DBP versus clinic DBP + nighttime DBP. *CV* cardiovascular, *BP* blood pressure, *SBP* systolic blood pressure, *DBP* diastolic blood pressure.

Table 2 shows BP levels of all participants during the baseline clinic visit. All BP phenotypes (clinic BP, 24-h BP, daytime BP, and nighttime BP) were higher in those who were defined as cases compared to controls. The correlations (r) of clinic BPs with 24-h BPs (both SBP and DBP) were 0.75 (*p* < 0.0001). Both clinic and 24-h SBP phenotypes were predictive of all-cause mortality along with daytime and nighttime DBP using logistic regression analyses. Using cox proportional hazard model, clinic and nighttime systolic and diastolic BPs were able to predict time to events (Hazard ratio estimates between 1.68 and 1.95, all *p* values < 0.02). Forrest Plot in Fig. 1 shows odds ratio of composite CV/renal disease outcomes comparing clinic, 24-h, daytime, and nighttime BP phenotypes, showing similar predictive abilities of clinic and 24-h BPs, with 10 mm Hg increments (actual odds ratios are shown in Supplemental Table 3). However, compared to the models with clinic BPs, likelihood ratio tests indicate that further adding, one at a time, 24-h BP phenotypes can improve the model fit for predicting the composite CV/renal disease events (24-h SBP [*p* = 0.002], 24-h DBP [*p* = 0.001], daytime SBP [*p* = 0.007], daytime DBP [*p* = 0.01], nighttime SBP [*p* = 0.04]) but not vice versa. One exception was nighttime DBP (*p* = 0.20) whose addition was not significant in predicting CV/renal events over clinic BPs. To directly compare predictivity of the 24-h BP phenotypes versus clinic BPs, we calculated area under the ROC curve (AUC) for each of the BP phenotypes. AUCs were slightly larger for 24-h BP phenotypes than clinic BP phenotypes (Fig. 2A–F). To further analyze how much more value 24-h BP phenotypes add to clinic BPs in predicting one CV/renal disease event, we calculated the average predicted probabilities of having an event for each of the BP phenotypes (Table 3, Supplemental Fig. 1A–B). In all participants, clinic BP measurements provided a slightly higher average probability of predicting an event than using 24-h BP phenotype (equivalent to 1 more event in 333 predictions), i.e. using clinic SBP (or DBP) would predict one more event than using 24-h SBP (or DBPs in 333 predictions). Using daytime SBP (or DBP) gives the same predictive probabilities as using the clinic SBP (or DBP). Using nighttime SBP we would predict 1 more event than using clinic SBP in 1,000 measurements, while nighttime DBP will predict 1 fewer event than using clinic DBP in 333 measurements.

**Table 3:** Average predictive probability of CV/renal event and comparison of predictions of various BP phenotypes.

BP variables	Average predicted probability in all-participant cohort	Number of predictions needed to make a difference of one predicted event
Clinic SBP	0.186	
24-h SBP	0.183	1 in 333^a^
Daytime SBP	0.186	Infinity
Nighttime SBP	0.187	1 in 1000^b^
Day–nighttime SBP difference	0.187	1 in 1000^b^
Clinic DBP	0.186	
24-h DBP	0.183	1 in 333^a^
Daytime DBP	0.186	Infinity
Nighttime DBP	0.183	1 in 333^a^
Day–nighttime DBP difference	0.183	1 in 333^a^

*CV* cardiovascular, *BP* blood pressure, *SBP* systolic blood pressure, *DBP* diastolic blood pressure.

^a^One more prediction when compared to clinic BP.

^b^One less prediction when compared to clinic BP.

The findings did not differ when only participants not on anti-hypertensive medications were analyzed (see supplemental data). The results also did not differ when we analyzed the data after adjusting the BP levels for anti-hypertensive medications (also shown in supplemental data).

### Nocturnal BPs

 Overall, African American subjects in our study showed less than 10% nocturnal dip. The magnitude of the nighttime BP dip did not differ between participants who experienced a CV/renal disease event and those who did not (Fig. 1). Day-nighttime differences were not predictive of composite CV/renal disease events or all-cause mortality . The odds ratio for predicting a CVD/renal disease event based on nighttime BP was no more robust than predictions based on other 24-h BP phenotypes (Supplemental Tables 3 and 5).

## Discussion

In this study of an African American cohort, we found that both outpatient clinic BPs and 24-h BP phenotypes are predictive of long-term composite CV/renal disease morbidity and mortality. Moreover, measurement of 24-h BP phenotypes provided limited incremental value as a predictor of CV/renal disease risk among African Americans, by differing in prediction by one less or more event in ~ 250 (range 250–1,000 depending on the 24-h BP phenotype) predictions, compared to clinic BP values. Even though several similar studies comparing clinic and 24-h BPs have been reported in Caucasian and Asian populations^24,25^, this is the first study in African Americans that directly compared the predictive effects of outpatient BP levels versus 24-h BP phenotypes in predicting CV/renal disease events^32^.

There has been no question of superiority of ABPM levels in improving the accuracy, minimizing the error, and standardization of measurements due to usage of automated devices in both African Americans and Caucasians^33,34^. ABPM is also essential to the diagnosis of various HTN phenotypes including white-coat, masked, and/or nocturnal in addition to BP variability^4,9,14,16^. With ABPM, it is also possible to assess the efficacy of anti-hypertensive medications throughout the day and night rather than at a single time-point with measurements of BPs in the office^35,36^.

Starting in early 1980s, several thousands of patients with ABPM have been followed for varying durations of time resulting in the conclusion that levels of ABPM phenotypes (among them nocturnal HTN) are better predictors of subclinical organ damage and CVD morbidity and mortality than the corresponding clinic BP values^17,19,21,22,24,25^. Some studies, but not all, have also associated 24-h BP levels with all-cause mortality^18,30,37–39^. Several prospective studies have addressed the CVD risk associated with white-coat HTN and indicate that both are associated with heightened risk^40^. Some recent studies have questioned this heightened risk with white-coat HTN and suggest that it may not be higher than their normotensive control subjects after adjustment of other risk factors^41–43^. In addition, masked HTN has been associated with increased CVD risk factors^43–45^. Most of these studies have been conducted in European and Asian populations^32^. In addition, ABPM levels are also valuable in evaluating BP variability which has been associated with target-organ damage^46,47^.

Few studies have evaluated the effect of ABPM on long-term CV/renal disease morbidity and mortality in African American individuals when compared to clinic BP values^32^. Most notable of this is Jackson Heart Study (JHS), which has shown that various 24-h BP phenotypes such as lack of nocturnal dipping, BP variability, and masked HTN were negatively associated with intermediate target-organ damage markers such as glomerular filtration rate decline (chronic kidney disease), increased left ventricular mass index, and increased carotid artery intimal-media thickness when controlled for clinic BPs indicating superiority of ABPM in predicting these intermediate outcomes^48–53^. In a recent study, JHS reported increased predictability of day time and night time BPs in prediction of CV events and all-cause mortality, however, they did not report on the incremental advantage bestowed by cumbersome 24 h BP measurements and how many events would be predicted by utilizing 24 h BP phenotypes^54^.

In this study, we compare the predictability of composite CV/renal disease outcomes and all-cause mortality by clinic BPs versus 24-h BP phenotypes among African Americans. While we found that all BP phenotypes were predictive of long-term outcomes, the incremental value of 24-h BP phenotypes over clinic BP measurements was limited and the average predictive difference was one less or more predicted CV/renal disease event in 250 individuals (range 250–1,000) compared to clinic BPs. Our findings are in contrast to findings from several studies that indicated that 24-h BPs indeed add a significant value over clinic BPs and save lives. However, almost all of these studies that have evaluated morbidity and mortality data were conducted in Caucasian and Asian subjects. Among studies in African American subjects, the findings are from evaluation of intermediate target-organ damage parameters rather than actual CV/renal disease events and in those that reported this data—the incremental advantage was not specified. In a recent publication based on JHS participants, a total of 165 events (including CVD events [n = 80] and all-cause mortality [n = 85]) were identified after a median 10.8-year follow-up among individuals who underwent ABPM. Five-year predicted probability of a CVD or an all-cause mortality event (combined) in this study was associated with increasing levels of clinic BPs, however, no comparison was made with ABPM phenotypes in this study^55^. Another study on masked HTN showed that CVD risk for masked HTN was not heightened compared clinic BPs in African Americans; C-statistic for CVD risk with masked HTN and clinic BPs being 0.681 and 0.703 respectively (higher the C-statistic, better the prediction model)^56^. Most recent study from JHS showed that only nighttime SBP was associated with modest increase in all-cause mortality^54^. In addition, hazard ratios for cardiovascular events was increased only with large BPs differences (10–16 mmHg) between 24 h phenotypes and clinic phenotypes^54^. These data from JHS and findings from our study suggest 24-h BP phenotypes may not be much superior to clinic BPs in predicting CV/renal disease events among African Americans.

An explanation for these findings could be due to higher 24-h BP levels seen among African Americans compared to Caucasians and Asians, particularly lack of day-night dip. Levels of ABPM phenotypes (24-h, daytime, and nighttime) are typically lower than clinic BP values with a clinic BP value of 140/90 mmHg approximately corresponding to a 24-h BP of 130/80 mmHg, daytime BP of 135/85 mmHg, and nighttime BP of 120/70 mmHg, values determined based on several studies^33^. However, the JHS found that outcome-derived ABPM thresholds for African Americans were higher than those from published recommendations for European, Asian, and South American populations with a clinic SBP ≥ 140 mmHg corresponding to 24-h, daytime, and nighttime SBP of 138 mmHg, 134 mm Hg, and 129 mmHg respectively^55^. We found similarly attenuated differences between clinic and 24-h BP phenotypes in our study population. As an example, albeit the measurements were separated by a few days, clinic BP value of 127/83 mmHg corresponded to 24-h BP of 124/83 mmHg, daytime BP of 124/75 mmHg, and nighttime BP of 120/71 mmHg among cases (Supplemental Table 4). It is plausible that this smaller difference between clinic and 24-h BP phenotypes negated whatever advantage was conferred by 24-h BPs in other ethnic groups.

In several racial/ethnic populations, nighttime BP has been shown to have the strongest prognostic value for CVD, compared with 24-h, daytime, and clinic BPs^22,57,58^. It is well-known that, compared with other ethnic groups, African Americans have a blunted nighttime BP dip^27,28^. While there are no outcome studies associating nocturnal HTN or non-dipping patterns with CV/renal disease morbidity and mortality, nocturnal BPs were shown to be associated with some intermediate markers of target organ damage among African Americans^49^. In the current study, we found that the nocturnal BP dip did not differ between cases and controls, and there was no evidence of superiority of nocturnal BP compared to other 24-h BP phenotypes and only provided modest improvement over clinic BPs as a predictor of CV/renal disease risk.

There are several strengths to this study. All CV/renal disease events were verified and adjudicated by reviewing patient medical records by board-certified physicians. In addition, medical records of all individuals who were determined to have not had an event for 10 years were also reviewed prior to designating them as controls unlike many of the other studies that have only verified positive events^32^. Despite its strengths, we acknowledge our study’s limitations. Our sample size is moderate. We do not have reliable information on history of smoking, alcohol use, or physical activity. Our definition of daytime and nighttime are based on fixed-clock times, which by including transitional times—the hours during which some patients may be asleep, whereas others are awake—may represent a source of mis-classification. However, studies have shown that the day and night durations defined by wide fixed-clock intervals (6:00 am to 10:00 pm for the day and 10:00 pm to 6:00 am for the night), narrow fixed-clock intervals (10:00 am to 8:00 pm for the day and midnight to 6:00 am for the night) or according to patient’s diary does not affect predictability of in-study outcomes^59^. Other limitations include measurement of 24-h BP levels were conducted in an in-patient facility rather than during their daily activities. Based on our BP levels which are comparable to what was reported to JHS, these limitations did not adversely affect the BP measurements.

Based on extensive reviews of the available data, European and American guidelines recommend that ABPM be considered an “adjunct”” to the “gold standard” clinic measurement^7,60^. While the value of ABPM in diagnosing and monitoring BP is undisputable, its real utility is in being a better predictor of CV/renal disease compared to clinic BPs. However, we found that 24-h BP phenotypes are only marginally better in predicting CVD events compared to clinic BPs among African Americans with 24-h BPs predicting one less or more CV/renal event in 250 individuals (range 250–1,000). It is plausible that this smaller difference compared to Asian and European populations is due to less significant differences between the levels of clinic BPs and 24-h BP phenotypes in African Americans. This raises questions of cost-effectiveness of ABPM and added burden of 24-h BP measurement among African Americans. In conclusion, in middle aged African Americans, our results suggest that a carefully measured clinic BP is nearly as effective as 24-h BP measurement in predicting CV/renal disease events and ABPM is of limited incremental value. Larger studies in African Americans are needed to confirm these findings.

## Data availability

All data will be provided in excel spreadsheet without restriction upon request.

## Supplementary information

Supplementary file1 (DOCX 423 kb)
