# Supplementary material for: Twenty-four-hour versus clinic blood pressure levels as predictors of long-term cardiovascular and renal disease outcomes among African Americans
Source: Sci Rep. 2020 Jul 15;10:11685. doi: 10.1038/s41598-020-68466-5 (PMC7363933; doi:10.1038/s41598-020-68466-5)
Supplement: Supplementary file 1 — Supplementary file1 (DOCX 423 kb) [file 41598_2020_68466_MOESM1_ESM.docx]

**Supplemental data for :**

**Twenty-four-hour versus clinic blood pressure levels as predictors of long-term cardiovascular and renal disease outcomes among African Americans**

^1^Srividya Kidambi, MD, ^1^Tao Wang, PhD, ^1^Thomas Chelius, MS, ^1^Irene Nunuk, MD, ^1^Priyanka Agarwal, MD, ^1^Purushottam Laud, PhD, ^1^David Mattson, PhD, ^1^Allen W. Cowley, Jr. PhD, ^1^Mingyu Liang, PhD, ^1^Theodore Kotchen, MD

^1^8701 Watertown Plank Road, Medical College of Wisconsin, Milwaukee, WI, USA

**No anti-hypertensive cohort:** Approximately 186 participants (23 cases, 163 controls) who were not on medications for HTN at the time of baseline clinic visit were included in this cohort (a sub-set of the all-participant cohort). Unadjusted baseline characteristics and BP levels of participants taking and not-taking anti-hypertensives at the baseline clinic visit are shown in **Supplemental Table 3**. Participants on medications were older, predominantly women, and followed for a longer period of time. After adjustment for age and sex, participants on anti-hypertensives had higher levels of BMI, total and LDL-cholesterol, and insulin and lower levels of HDL-cholesterol. PRA levels were no longer significant after adjustment of age and sex. **Supplemental Table 5** shows BP levels in this group. All-cause mortality was predicted by clinic BPs but not by any of the 24-hour BP phenotypes in this cohort **(Supplemental Table 6)**. Prediction of composite CV/renal disease outcomes was similar to the all-participant cohort (**Supplemental Table 6)** with all BP phenotypes being significantly predictive. Comparing clinic and 24-hour BPs, 95% confidence intervals for the odds ratios for CV/renal disease events overlapped **(Supplemental Figure 2)**. However, after adjustment for clinic BP, 24-hour BP phenotypes resulted in a statistically significant increment in predictability but to a much lesser degree than when the all-participant cohort was analyzed (24-hour SBP [p=0.02], 24-hour DBP [p=0.03], daytime SBP [p=0.04], and nighttime SBP [p=0.02], but not daytime DBP [p=0.08] or nighttime DBP [p=0.12]). Areas under curve (AUC) for 24-hour BP phenotypes and clinic BP phenotypes **(Supplemental Figures 3A-F)** were also similar. As in the all-participant cohort, using clinic BP gave a higher average probability of predicting an event than using 24-hour BP phenotypes (1 more event in 250 predictions) **(Supplemental Table 7)**. Using daytime SBP (or DBP) predicts one less event in 500 measurements compared to using the clinic SBP (or DBP). Using nighttime SBP, we would predict 1 more event than using clinic SBP in 1000 measurements, while nighttime DBP will predict 1 less event than using clinic DBP in 200 measurements.

**All-participant cohort with adjusted clinic BPs:** Based on previously published reports, stepped increments of 8/4 mmHg, 14/10 mmHg, and 20/16 mmHg were added to measured systolic and diastolic BPs of treated subjects taking 1, 2, and ≥ 3 drug classes, respectively to obtain adjusted BP levels^31,32^. The mean adjusted systolic and diastolic BPs are shown in **Supplemental Table 8**. Adjusted Odds ratios for composite CV/renal disease outcomes for adjusted clinic BP phenotype are shown in **Supplemental Table 4**. After adjustment for adjusted clinic BPs, 24-hour BP phenotypes still resulted in slight but statistically significant increments in the predictability of a composite CV/renal disease event (24-hour SBP [p=0.008], 24-hour DBP [p=0.006], daytime SBP [p=0.02], daytime DBP [p=0.049], but not with nighttime SBP [p=0.07] or nighttime DBP [p=0.47]. ROC statistics are shown in **Supplemental Figures 4A-F.**

Supplemental Table 1: Epigenetics of Hypertension- Population Study - Cardiovascular Disease, Renal Disease, and Death Adjudication Criteria

Primary Reviewer: ____________________ Review Date: __________________

Second Reviewer: _______________________ Review Date: __________________

Adjudicator: _______________________ Review Date: __________________

**Cardiovascular disease:**

1. Myocardial infarction: Any 2 out of 1-3, one out of 5-8* or one out of 4 or 9. Excludes unstable angina and stimulant (cocaine) induced MI, and excludes procedure-associated MI.
2. ECG pattern
   1. ST- T abnormalities: depression or elevation >1mm
   2. Pathologic Q wave >0.04 second duration
   3. New LBBB
3. Cardiac enzyme elevation
   1. Troponin > 2x upper limit normal
   2. Troponin > upper limit normal but < 2x upper limit
   3. CK-MB index elevation
   4. CK-MB > 2x upper limit normal
   5. CK-MB > upper limit normal but < 2x upper limit
   6. CPK > 2x upper limit normal
   7. CPK > upper limit normal but < 2x upper limit
4. Physician documentation of cardiac chest pain: a and b
   1. Chest, jaw, throat or arm pain, discomfort or tightness of at least 15 minutes
   2. Excludes definite non-cardiac causes of chest pain
5. Autopsy/death certificate that reports underlying cause of death as:
6. MI
7. Problem list in EMR*
8. Past medical history in EMR*
9. Care Everywhere in EMR*
10. Discharge summary*
11. ICD9/10 code

* With accompanying documentation of MI in a cardiologists’ s note

1. Aneurysm and dissection of heart: AAA, aortic dissection and coronary aneurysm?
2. AAA on imaging including ultrasound, CT, MRI or angiogram
3. History of surgical or endovascular repair of AAA
4. Cardiac arrest*

* Excluding drug-induced, pulmonary embolism related events

1. Congestive heart failure (not iatrogenic) requiring hospitalization*
2. LV systolic dysfunction with symptoms in (4)
3. LV diastolic dysfunction with symptoms in (4)
4. CXR showing cardiomegaly or pulmonary edema/congestion with symptoms in (4)
5. Orthopnea, elevated JVP, elevated CVP, edema and volume overload on exam, exertional dyspnea, weight gain
6. Diagnosed by physician, received treatment for CHF (including diuretic, ACEI, ARB, beta blocker)

* Excluding alcoholic, viral infection related cardiomyopathy, non-ischemic cardiomyopathy in a non-hypertensive.

1. Coronary revascularization
2. CABG
3. PTCA
4. Coronary angioplasty
5. Thrombolytic agent
6. Atherectomy
7. Peripheral vascular disease
8. Surgery, angioplasty, or thrombolytic agent for peripheral revascularization
9. History of amputation due to ischemia/peripheral vascular disease
10. Carotid angioplasty with stent
11. Carotid endarterectomy
12. Stroke: All of 1-3, one out of 4 or 9 or one out of 5-8*. Excludes TIA which is defined as rapid onset neurologic deficit lasting >30 seconds to <24 hours. Excludes procedure induced stroke.
13. Rapid onset neurologic deficit must last more than 24 hours except in case of death or revascularization
14. Deficit not due to brain trauma, tumor, infection (encephalitis/abscess), metabolic abnormalities, seizures or other cause
15. Correlating imaging evidence on CT, MRI, CTA or MRA. Or correlating blood on lumbar puncture
16. Autopsy/Death certificate
17. Problem list
18. Medical history
19. Care Everywhere
20. Discharge summary
21. ICD9/10 code

*With accompanying documentation of CVA in a neurologists’ s note

*Etiology of stroke*:

1. Ischemic (including ischemic infarction with transformation to hemorrhagic later)
   - - - 1. Cardio embolic
         2. Small vessel disease
         3. Large vessel disease
         4. Thrombosis
         5. Embolus
2. Hemorrhagic
3. Other
4. 2 or more causes
5. Negative evaluation
6. Incomplete evaluation

Comments after each section

**renal disease***

1. **CKD stage V**: GFR of 15 or less not on dialysis
2. **ESRD/kidney dialysis**: permanent requirement of hemodialysis or peritoneal dialysis
3. **Kidney transplant**

Etiology for transplant

* Excluding non-hypertensive renal events

**Death**

Date of death if known: **_______________________**

Materials available (Mark all that apply):

1.1 Death certificate

1.2 Hospital records

1.3 EMS report

1.4 Exit interview

1.5 Autopsy findings

1.6 Other:

Review death certificate, records, and/or autopsy (if available) for the following causes:

1. Definite, probable or possible MI
2. Heart failure
3. Stroke
4. Ruptured aortic aneurysm
5. ESRD

6) None of the above: In that case, is the death related to the following

1. Other cardiac (e.g. myocarditis)
2. Cancer
3. Accident/Injury/Suicide/Homicide
4. Liver disease
5. Infection
6. Dementia
7. Chronic lung disease
8. Pulmonary embolism
9. Other non-cardiac, non-stroke death. ________________________________________

Contributory causes of death (Mark all that apply):

1. Stroke (i) Cancer
2. MI (j) Sepsis
3. CHF (k) Alzheimer’s disease
4. Chronic liver disease and cirrhosis (l) Parkinson’s disease
5. Chronic lower respiratory diseases (m) suicide
6. Accidents (n) Homicide
7. Diabetes (o) Pulmonary embolus
8. Influenza or pneumonia (p) CKD

Other: ___________________ _____________________ _______________________

Supplemental Table 2. Incident cardiovascular and renal disease events

| Event type | Number of events |
| --- | --- |
| **Total events** | **77** |
| Cardiac events (myocardial infarctions, cardiac arrest, cardiac revascularization, or congestive heart failure) | 35 (72%) |
| Cerebrovascular events (stroke) | 26 (52%) |
| Renal events (end-stage renal disease, hemodialysis, and/or renal transplant related to HTN) | 8 (16%) |
| Peripheral vascular disease | 5 (10%) |
| Aortic dissection | 3 (6%) |
|  |  |
| More than 1 event | 23 (46%) |
|  |  |
| Event type | Number of subjects |
| **Total subjects with events** | **50** |
| Number of subjects with more than 1 event | 13 (26%) |
| Cardiac events only | 14 (28%) |
| Cerebrovascular events only | 19 (38%) |
| Renal events only | 2 (4%) |
| Peripheral vascular disease only | 1 (2%) |
| Aortic dissection only | 1 (2%) |

Supplemental Table 3. Comparison of baseline characteristics of participants were on medication versus those who were not on medications at the initial clinic visit (unadjusted)

|  | On medications  (n= 84) | Not on medications  (n=186) |
| --- | --- | --- |
| Women (%)^¶^ | 55 (65%) | 93 (50%)^*^ |
| Hypertensive (%)^¶^ | 84 (100%) | 65 (35%)^****^ |
| Mean length of follow-up (years) | 16.7 ± 4.7 | 12.8 ± 3.1^****^ |
| Age (years) | 45 ± 7 | 42 ± 7^****^ |
| BMI (kg/m^2^) | 30.2 ± 5 | 28.0 ± 5^***^ |
| Total Cholesterol (mg/dL) | 194 ± 43 | 173 ± 34^****^ |
| LDL-C (mg/dL) | 127 ± 42 | 109 ± 32^**^ |
| HDL-C (mg/dL) | 44 ± 15 | 48 ± 18^*^ |
| Triglycerides (mg/dL) | 107 ± 50 | 93 ± 67 |
| Glucose (mg/dL) | 89 ± 13 | 91 ± 19 |
| Insulin (µIU/L) | 14.7 ± 6.1 | 11.6 ± 6.8^***^ |
| Creatinine (mg/dL) | 0.9 ± 0.2 | 0.9 ± 0.2 |
| Supine PRA (ng ⁄mL⁄ h) | 0.7 ± 0.8 | 1.1 ± 2.1^*^ |
| Standing PRA (ng ⁄mL⁄ h) | 1.2 ± 3.1 | 1.5 ± 2.5 |
| Supine aldosterone (ng/dL) | 5.1 ± 3.3 | 4.6 ± 3.7 |
| Standing aldosterone (ng/dL) | 8.0 ± 4.4 | 7.2 ± 5.3 |

SD: standard deviation, BMI: body mass index, HDL-C: high-density lipoprotein-cholesterol, LDL-C: low-density lipoprotein cholesterol (LDL-C), PRA: plasma renin activity

All values are expressed as mean ± SD unless otherwise specified

^¶^ Expressed as percentages

^†^ Inter-quartile range

^*^p <0.05, ^**^p ≤0.01, ^***^p ≤0.001, ^****^p ≤0.0001

Conversion to SI units (multiplication factor): total cholesterol, LDL-C, and HDL-C: 0.0259 (mmol/L), HDL-C, triglycerides: 0.0113 (mmol/L), glucose: 0.05555 (mmol/L), insulin: 6.945 (pmol/L), creatinine: 88.4 (µmol/L), PRA (pg/mL): 0.0237 (pmol/L), aldosterone: 27.74 (pmol/L).

Supplemental Table 4. Predictive value of composite CV/renal disease risk of various BP phenotypes expressed as odds ratios^¶^ (95% confidence intervals) in all-participant cohort

| **Blood Pressure** | **All-cause mortality** | **Composite CV/**  **renal disease outcome** |
| --- | --- | --- |
| Clinic SBP | 1.39 (1.12-1.72)^**^ | 1.47 (1.24-1.75)^****^ |
| Clinic DBP | 1.82 (1.33-2.49)^***^ | 1.75 (1.36-2.26)^****^ |
| Adjusted clinic SBP | 1.43 (1.16-1.76)^***^ | 1.41(1.13-1.74)^**^ |
| Adjusted clinic DBP | 1.84 (1.36-2.50)^****^ | 1.79(1.27-2.52)^***^ |
| 24-hour SBP | 1.39 (1.05-1.83)^*^ | 1.76 (1.40-2.22)^****^ |
| 24-hour DBP | 1.34 (0.89-2.01) | 2.47 (1.70-3.58)^****^ |
| Day-time SBP | 1.36 (1.03-1.80)^*^ | 1.70 (1.36-2.13)^****^ |
| Day-time DBP | 1.46 (0.97-2.20) | 2.16 (1.53-3.05)^****^ |
| Night-time SBP | 1.61 (1.19-2.17)^**^ | 1.52 (1.23-1.88)^****^ |
| Night-time DBP | 1.80 (1.18-2.73)^**^ | 1.59 (1.16-2.18)^**^ |
| Day-Night SBP | 0.58 (0.31-1.08) | 0.92 (0.60-1.39) |
| Day-Night DBP | 0.46 (0.19-1.15) | 1.32 (0.72-2.41) |

CV: cardiovascular, BP: blood pressure, SBP: systolic blood pressure, DBP: diastolic blood pressure

**^¶^**Odd ratios of composite outcome for every 10 mm Hg increase in BP

^*^p ≤0.05, ^**^p ≤0.01, ^***^p ≤0.001, ^****^p ≤0.0001

Supplemental Table 5: Blood pressure levels (mean ± SD) of participants not on anti-hypertensives (no anti-hypertensive cohort)

|  | Overall  (n=186) | Cases  (n=23) | Controls  (n=163) |
| --- | --- | --- | --- |
| Clinic SBP (mm Hg) | 127 ± 22 | 146 ± 22 | 125 ± 20^****^ |
| Clinic DBP (mm Hg) | 83 ± 15 | 95 ± 15 | 81 ± 14^****^ |
| 24-hour SBP (mm Hg) | 124 ± 16 | 139 ± 15 | 121 ± 15^****^ |
| 24-hour DBP (mm Hg) | 83 ± 15 | 83 ± 12 | 73 ± 10^****^ |
| Day-time SBP (mm Hg) | 124 ± 17 | 139 ± 16 | 122 ± 16^****^ |
| Day-time DBP (mm Hg) | 75 ± 11 | 83 ± 11 | 74 ± 11^***^ |
| Night-time SBP (mm Hg) | 120 ± 18 | 136 ± 17 | 118 ± 17^****^ |
| Night-time DBP (mm Hg) | 71 ± 12 | 78 ± 12 | 69 ± 12^**^ |
| Day-Night SBP (mm Hg) | 5 ± 7 | 3 ± 10 | 6 ± 7 |
| Day-Night DBP (mm Hg) | 5 ± 5 | 4 ± 6 | 5 ± 5 |
| Day-Night SBP difference (%) | 4.4 ± 6.1 | 2.1 ± 7.5 | 4.8 ± 5.8 |
| Day-Night DBP difference (%) | 6.3 ± 7.2 | 4.7 ± 7.4 | 6.5 ± 7.2 |
| Day-Night SBP ratio | 0.96 ± 0.06 | 0.98 ± 0.08 | 0.95 ± 0.06 |
| Day-Night DBP ratio | 0.94 ± 0.07 | 0.95 ± 0.07 | 0.93 ± 0.07 |

SD: standard deviation, SBP: systolic blood pressure, DBP: diastolic blood pressure

^*^p <0.05, ^**^p ≤0.01, ^***^p ≤0.001, ^****^p ≤0.0001

Supplemental Table 6. Predictive value of composite CV/renal disease risk of various BP phenotypes expressed as odds ratios^¶^ (95% confidence intervals) in participants not on anti-hypertensives (no anti-hypertensive cohort)

| **Blood Pressure** | **All-cause mortality** | **Composite CV/**  **renal disease outcome** |
| --- | --- | --- |
| Clinic SBP | 1.52(1.11-2.07) ^**^ | 1.41(1.14-1.75) ^**^ |
| Clinic DBP | 2.28(1.36-3.85) ^**^ | 1.79(1.27-2.53) ^***^ |
| 24-hour SBP | 1.11(0.72-1.70) | 1.78(1.29-2.45) ^***^ |
| 24-hour DBP | 1.19(0.63-2.28) | 2.38(1.46-3.88) ^***^ |
| Day-time SBP | 1.08(0.70-1.66) | 1.70(1.25-2.31) ^***^ |
| Day-time DBP | 1.26(0.65-2.45) | 2.16(1.36-3.44) ^***^ |
| Night-time SBP | 1.18(0.78-1.77) | 1.69(1.22-2.34) ^**^ |
| Night-time DBP | 1.17(0.64-2.13) | 1.79(1.11-2.90) ^*^ |
| Day-Night SBP | 0.44(0.13-1.49) | 0.45(0.21-0.97) ^*^ |
| Day-Night DBP | 1.00(1.10-2.03) | 0.74(0.24-2.28) |

CV: cardiovascular, BPO: blood pressure, SBP: systolic blood pressure, DBP: diastolic blood pressure

**^¶^**Odd ratios of composite outcome for every 10 mm Hg increase in BP

^*^p ≤0.05, ^**^p ≤0.01, ^***^p ≤0.001, ^****^p ≤0.0001

Supplemental Table 7. Average predictive probability of CV/renal event and comparison of predictions of various BP phenotypes

| BP variables | Average predicted probability in  no anti-hypertensive cohort | Number of predictions needed to make a difference of one predicted event |  |
| --- | --- | --- | --- |
| Clinic SBP | 0.124 |  |  |
| 24- hour SBP | 0.120 | 1 in 250^*^ |  |
| Daytime SBP | 0.122 | 1 in 500^*^ |  |
| Nighttime SBP | 0.125 | 1 in 1000^¶^ |  |
| Day-nighttime SBP difference | 0.125 | 1 in 1000^¶^ |  |
| Clinic DBP | 0.124 |  |  |
| 24- hour DBP | 0.120 | 1 in 250^*^ |  |
| Daytime DBP | | 0.122 | 1 in 500^*^ |
| Nighttime DBP | | 0.119 | 1 in 200^*^ |
| Day-nighttime DBP difference | | 0.119 | 1 in 200^*^ |

CV: cardiovascular, BP: blood pressure, SBP: systolic blood pressure, DBP: diastolic blood pressure

^*^one more prediction when compared to clinic BP

^¶^ one less prediction when compared to clinic BP

Supplemental Table 8: Clinic blood pressure levels (mean ± SD) in the all-participants after adjustment for treatment at baseline

|  | Overall  (n=270) | Cases  (n=50) | Controls  (n=220) |
| --- | --- | --- | --- |
| Adjusted clinic SBP (mm Hg) | 135 ± 24 | 154 ± 21 | 131 ± 23^****^ |
| Adjusted clinic DBP (mm Hg) | 88 ± 16 | 101 ± 15 | 85 ± 15^****^ |

SD: standard deviation, SBP: systolic blood pressure, DBP: diastolic blood pressure

^***^p ≤0.001, ^****^p ≤0.0001


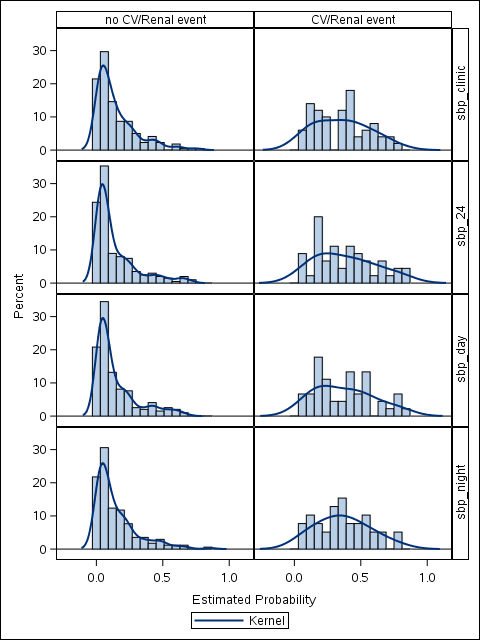


**Supplemental Figure 1A: Estimated probability of having a CV/renal disease outcome with different SBP phenotypes**

Predictive probabilities of each of the SBP phenotypes: sbp_clinic: clinic SBP, sbp_24: 24-hour SBP, sbp_day: daytime SBP, sbp_night: nighttime SBP

CV: cardiovascular, SBP: systolic blood pressure


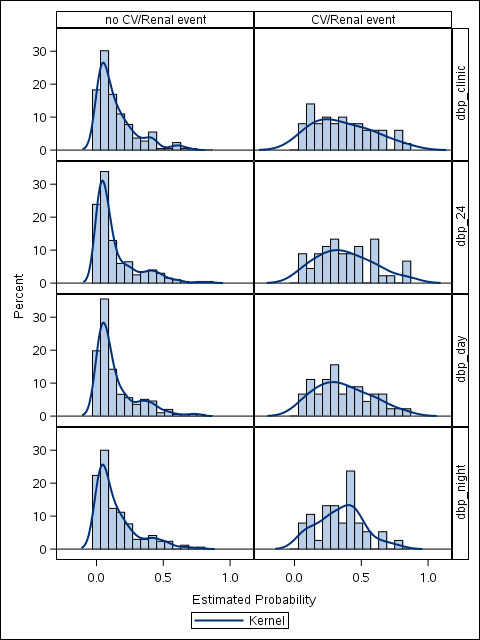


**Supplemental Figure 1B: Estimated probability of having a CV/renal disease outcome with different DBP phenotypes**

Predictive probabilities of each of the DBP phenotypes: dbp_clinic: clinic DBP, dbp_24: 24-hour DBP, sbp_day: daytime DBP, sbp_night: nighttime DBP

CV: cardiovascular, DBP: diastolic blood pressure

Supplemental Figure 2. Forest plot showing odds ratio (CI) for a composite CV/renal event with different clinic and 24-hour blood pressure phenotypes in the cohort not-taking anti-hypertensive medications

Odds ratio for compositie CV/renal events are shown for different blood pressure phenotypes are shown with overlapping CI in the cohort not-taking anti-hypertensive medications

CI: confidence interval, CV: cardiovascular, SBP: systolic blood pressure, DBP: diastolic blood pressure

Figures 3A-F. Receiver-operating Curves (ROC) for the prediction of composite CV/renal disease events (clinic BP vs. 24-hour BP phenotypes) in the cohort not-taking anti-hypertensive medications

**3E**

**3C**

**3A**


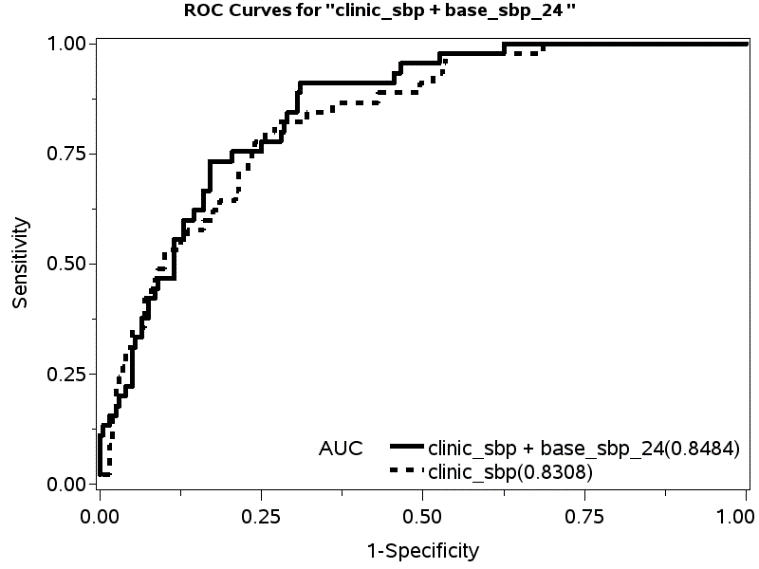

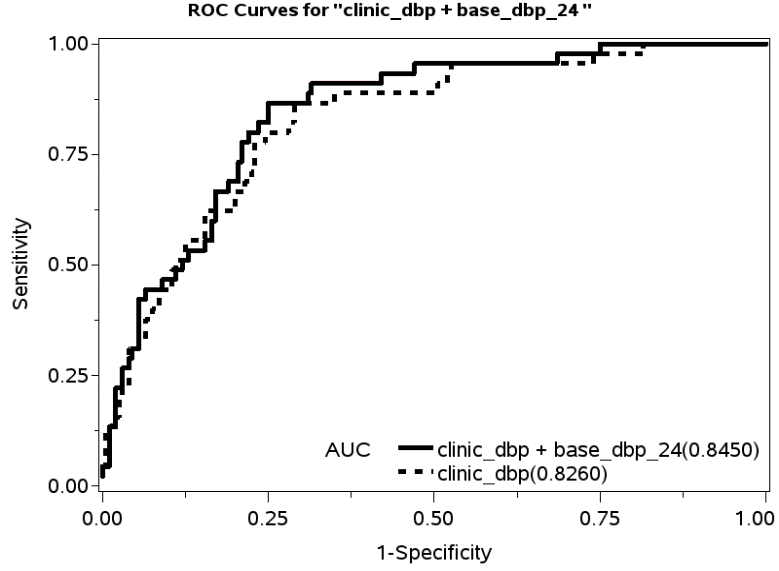


**3B**


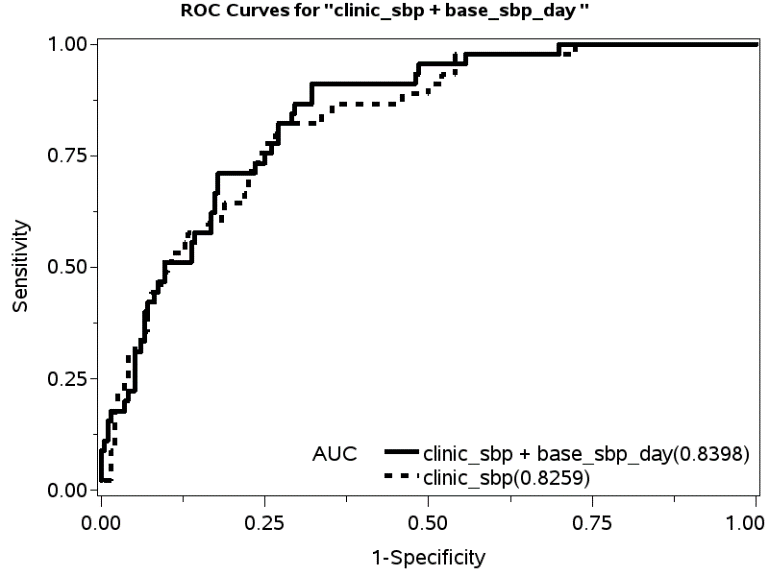

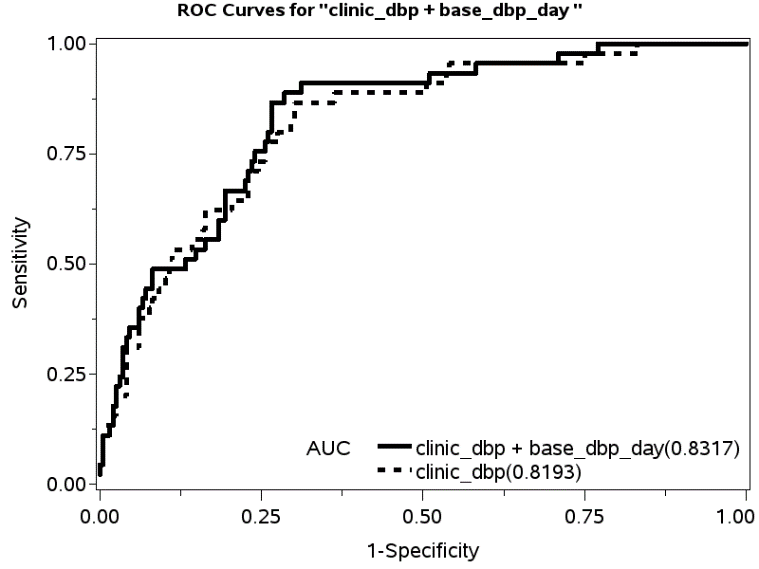


**3D**


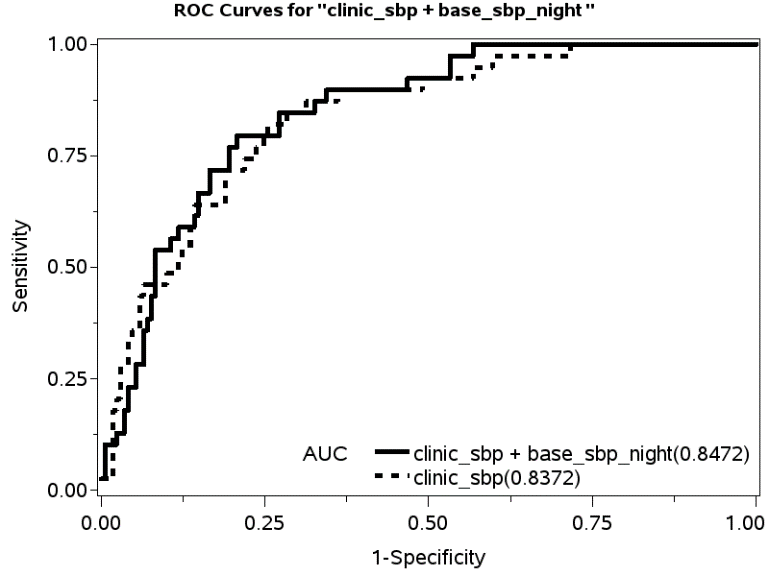

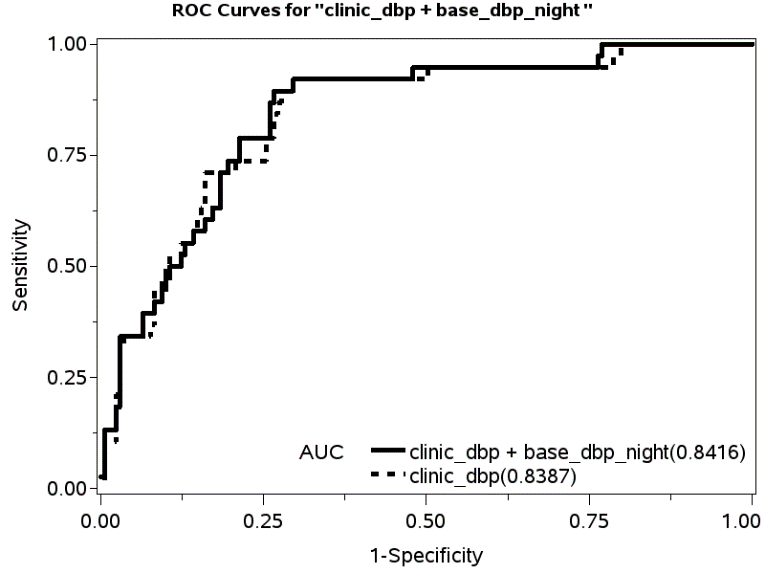


**3F**

3A: clinic SBP vs. clinic SBP + 24-hour SBP, 3B: clinic DBP vs. clinic DBP + 24-hour DBP, 3C: clinic SBP vs. clinic SBP + daytime SBP, 3D: clinic DBP vs. clinic DBP + daytime DBP, 3E: clinic SBP vs. clinic SBP + nighttime SBP, 3F: clinic DBP vs. clinic DBP + nighttime DBP

CV: cardiovascular, BP: blood pressure, SBP: systolic blood pressure, DBP: diastolic blood pressure

CI: confidence interval, CV: cardiovascular, SBP: systolic blood pressure, DBP: diastolic blood pressure


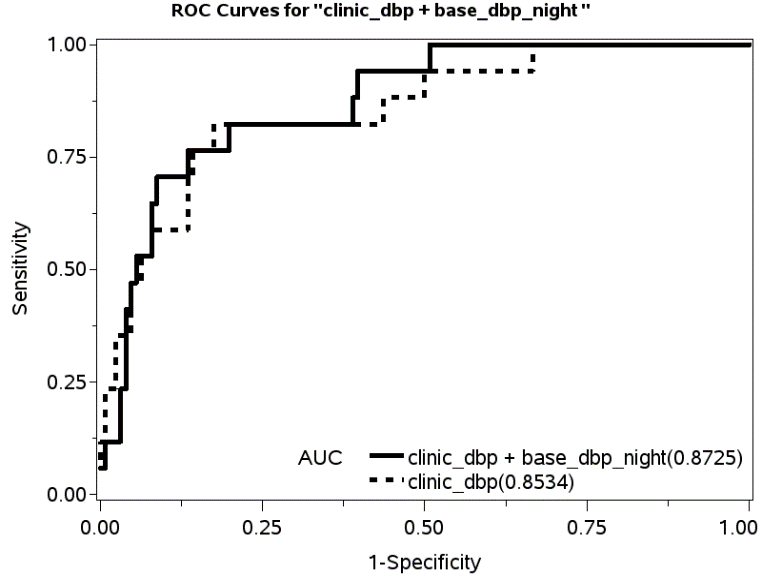

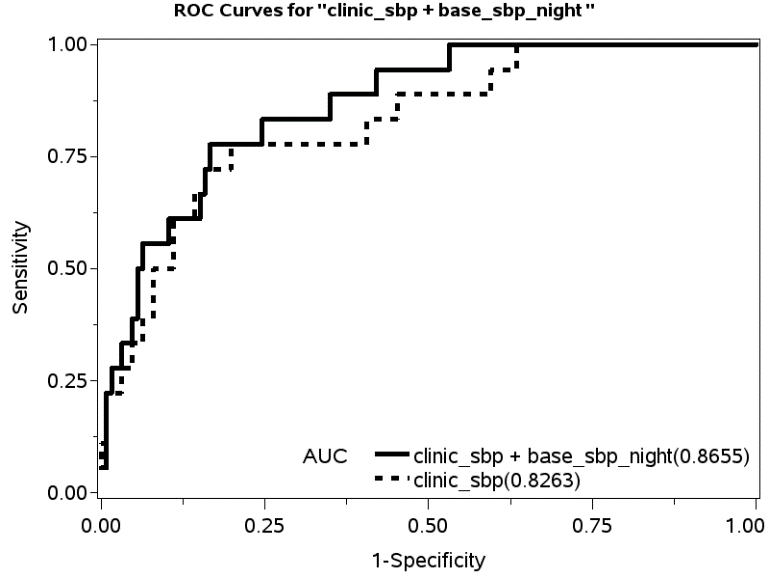

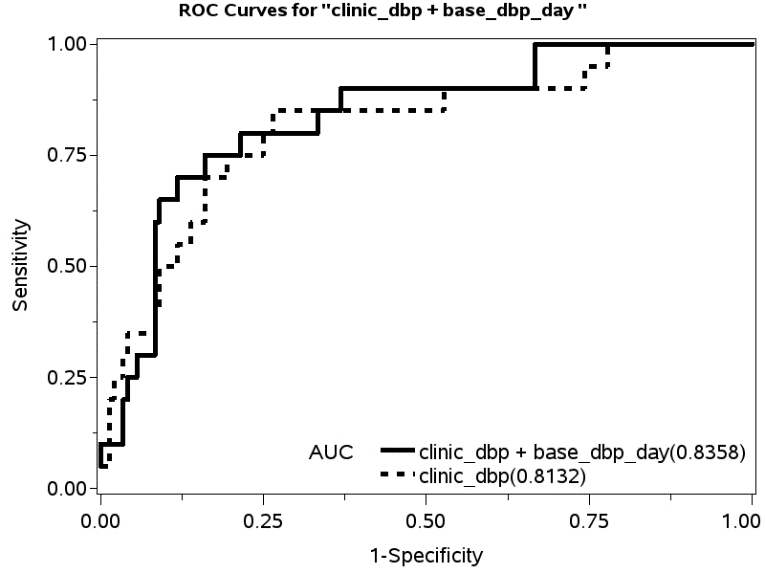

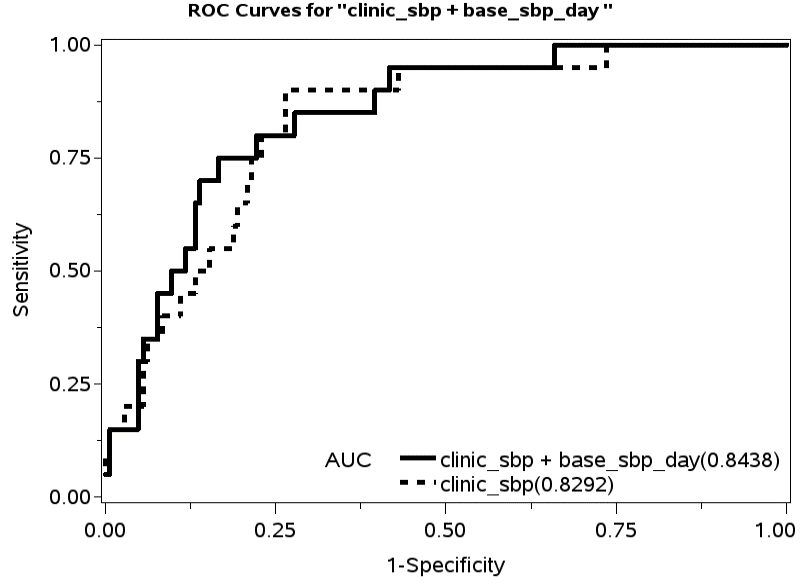

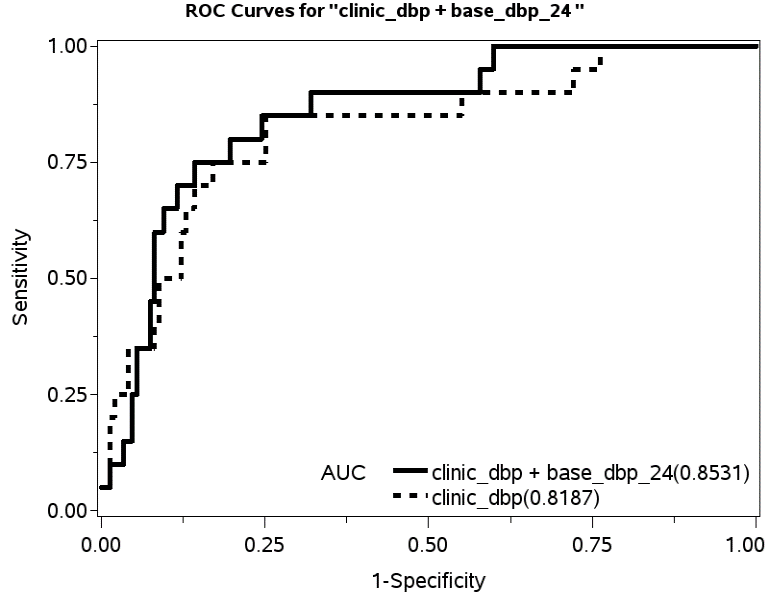

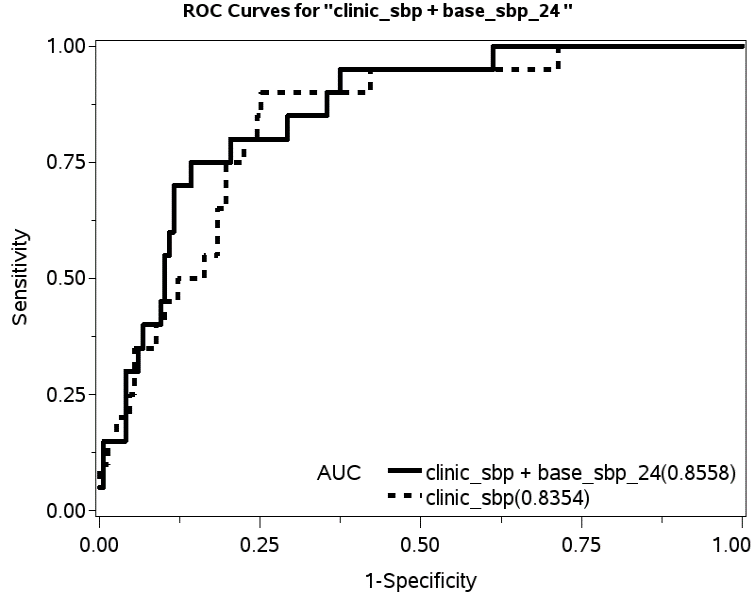
Supplemental Figure 4A-F. Receiver-operating Curves (ROC) for the prediction of composite CVD/renal disease events (adjusted clinic BP vs. 24-hour BP phenotypes) in all-participant cohort with adjusted clinic BPs

**2F**

**2B**

**2CA**

**2DA**

**2E**

**2A**

2A: adj. clinic SBP vs. adj. clinic SBP + 24-hour SBP, 2B: adj. clinic DBP vs. adj. clinic DBP + 24-hour DBP, 2C: adj. clinic SBP vs. adj. clinic SBP + daytime SBP, 2D: adj. clinic DBP vs. adj. clinic DBP + daytime DBP, 2E: adj. clinic SBP vs. adj. clinic SBP + nighttime SBP, 2F: adj. clinic DBP vs. adj. clinic DBP + nighttime DBP

adj.: adjusted, BP: blood pressure, SBP: systolic blood pressure, DBP: diastolic blood pressure
